# Regulation of Autophagy by Kinases

**DOI:** 10.3390/cancers3022630

**Published:** 2011-06-09

**Authors:** Savitha Sridharan, Kirti Jain, Alakananda Basu

**Affiliations:** Department of Molecular Biology and Immunology, Institute for Cancer Research, University of North Texas Health Science Center, Fort Worth, TX 76107, USA; E-Mails: ssridhar@live.unthsc.edu (S.S.); kjain@live.unthsc.edu (K.J.)

**Keywords:** autophagy, protein kinase, mTOR, p70S6K, AMPK, PI3K, Akt, MAPK, PKC

## Abstract

Autophagy is a process of self-degradation that maintains cellular viability during periods of metabolic stress. Although autophagy is considered a survival mechanism when faced with cellular stress, extensive autophagy can also lead to cell death. Aberrations in autophagy are associated with several diseases, including cancer. Therapeutic exploitation of this process requires a clear understanding of its regulation. Although the core molecular components involved in the execution of autophagy are well studied there is limited information on how cellular signaling pathways, particularly kinases, regulate this complex process. Protein kinases are integral to the autophagy process. Atg1, the first autophagy-related protein identified, is a serine/threonine kinase and it is regulated by another serine/threonine kinase mTOR. Emerging studies suggest the participation of many different kinases in regulating various components/steps of this catabolic process. This review focuses on the regulation of autophagy by several kinases with particular emphasis on serine/threonine protein kinases such as mTOR, AMP-activated protein kinase, Akt, mitogen-activated protein kinase (ERK, p38 and JNK) and protein kinase C that are often deregulated in cancer and are important therapeutic targets.

## 1. Introduction

Cell death is vital to the proper functioning, development and homeostasis in an organism. The ability of cells to evade cell death can lead to cancer. Programmed cell death is a complex series of well-orchestrated events that result in the controlled demise of the cell. Apoptosis or type I programmed cell death is characterized by the activation of proteases that results in the destruction of cellular components and cell shrinkage facilitating its phagocytosis. Regulated necrosis or type III programmed cell death is a series of ordered events that lead to a more explosive form of death [1].

Autophagy, a process of “self-eating”, is often activated in response to starvation and stress [2]. Several forms of autophagy including macroautophagy, mitophagy, reticulophagy and pexophagy have been reported [3]. Macroautophagy (often referred to as autophagy) is characterized by the engulfment of cell's own cytoplasm and organelles in double membrane vesicles resulting in lysosomal degradation of the contents [2]. The autophagic degradation products serve as raw materials for cellular metabolism to generate energy in order to survive cellular stress and nutrient deprivation [4]. Under normal conditions, basal autophagy functions to remove aged and damaged organelles and proteins [5]. Excessive autophagy may, however, lead to death referred to as type II programmed cell death [1].

Autophagy has been regarded as a survival mechanism since it provides nutrient deprived cells with a means of survival. However, autophagy has been regularly observed in dying cells [6]. This raises the question as to whether autophagy is indeed a mode of cell death or if it is simply a failed attempt to rescue stressed cells from death [6]. Also, there is extensive cross-talk between autophagy and other forms of cell death, such as apoptosis and necrosis. Autophagy can precede, inhibit or enhance apoptotic cell death and both autophagic and necrotic morphologies are observed in the same cell [6]. Conversely, autophagy inhibition can sensitize cells to necrosis [7]. Hence, the outcome of autophagy on cell death may also depend on its cross-talk with apoptotic and necrotic pathways.

The process of autophagy involves a series of steps, including induction, cargo recognition and selection, vesicle formation, autophagosome-lysosome fusion, digestion of cargo and finally release of digestion products into the cytosol [8]. Autophagy is well established in yeast and involves several proteins known as autophagy-related or Atg proteins. The first known autophagy protein in yeast is Atg1, a serine/threonine kinase [9]. Atg1 forms a complex with Atg13 and Atg17 and initiates the formation of autophagosomes [10]. The mammalian homologs of Atg1 are ULK1 and -2 (Unc-51-like kinase 1 and -2) and that of Atg17 is FIP200 (the focal adhesion kinase family-interacting protein of 200 kD) [11]. ULKs and FIP200 form a complex with mammalian Atg13 which translocates to phagophore upon starvation and mediates autophagy induction [12]. During nutrient-rich conditions, Atg1 is inhibited by another serine/threonine kinase TOR (target of rapamycin) [13]. The recognition of a particular cargo protein, preApe1 in yeast, is mediated by a receptor protein Atg19, which subsequently interacts with Atg8 to mediate packaging of preApe1 into transport vesicles [14-16]. In the mammalian system, p62/SQSTM1 (sequestosome 1) is involved in the recognition of ubiquitinated cargo proteins. It binds to LC3 (microtubule-associated protein 1 light chain 3), which is the mammalian ortholog of Atg8 and recruits polyubiquitinated protein aggregates to the autophagosome for degradation [17,18]. The phagophore membrane initiation is mediated by class III phosphatidylinositol-3 kinase (PI3K) or Vps34 (vacuolar proteins sorting 34) which forms complex with Vps15 (p150), Atg14 (Barkor) and Atg6 (beclin 1)/Vps30 [19-21]. In unstressed conditions, beclin 1 remains inhibited by the interaction with the antiapoptotic protein Bcl-2 [22]. Dissociation of beclin 1 from Bcl-2 is required for the induction of autophagy [23]. The PI3K complex recruits other Atg proteins, such as Atg 18, 20, 21 and 24 to the pre-autophagosomal membrane which subsequently aid in the recruitment of two ubiquitin-like conjugating systems, namely Atg12-Atg5-Atg16 and Atg8-phosphatidylethanolamine to mediate the expansion of autophagosome [24-27].

Several studies point to the tumor suppressive functions of autophagy. Beclin 1 is a haploinsufficient tumor suppressor [28,29]. It has been reported that *Beclin1*, and *Atg7* are deleted in several cancers [30]. Moreover, well-studied tumor suppressor proteins such as PTEN induce autophagy whereas oncogenes inhibit it [31]. Also, the accumulation of p62 as a result of defective autophagy has been shown to contribute to tumorigenesis [32]. However, the role of autophagy in nutrient recycling has led to the belief that it provides tumor cells with the ability to survive stress. Consistent with this notion are several reports that suggest a role for autophagy in tumorigenesis and tumor progression. For example, autophagy has been shown to cause resistance to chemotherapeutic drugs [33], mediate anti-estrogen resistance [34] and resistance against detachment-induced cell death in breast cancer [35]. It is believed that chronic suppression of autophagy may stimulate oncogenesis but once tumor initiation has occurred it may play a protective role against cancer therapy by enabling tumor cells to overcome stress [31]. Thus, autophagy has been considered a double-edged sword in cancer [36].

Given the ambiguous role of autophagy in cancer, there is a growing interest in the pathways and molecules that regulate it. Kinases play an integral role in the inception and execution of autophagy. While some kinases such as mTOR, PI3K and AMPK directly regulate components of autophagic machinery, the precise role of other kinases such as mitogen-activated protein kinase (MAPK) and protein kinase C in autophagy is less well characterized and they regulate autophagy indirectly by the modulation of the levels/function of autophagy related proteins. The purpose of this review article is to discuss current literature on the role of several kinases in autophagy regulation that has important implication in cancer and other diseases.

## 2. mTOR

The mammalian target of rapamycin (mTOR) is a serine/threonine kinase that belongs to the phosphatidylinositol kinase-related kinase (PIKK) family [37]. It was first described as the physiological target of the immunosuppressant drug rapamycin [38]. Subsequent studies established its role in protein translation and cell growth [39]. Owing to its energy sensing functions, mTOR is considered the master regulator of autophagy [40]. mTOR forms two complexes, mTORC1 (mTOR, raptor, mLST8 and PRAS40) and mTORC2 (mTOR, rictor, mLST8 and Sin1) which differ in composition as well as functions [41,42]. mTORC2 is involved in the regulation of several AGC kinases such as Akt and protein kinase C (PKC) [43-45] whereas mTORC1 acts via its downstream targets 40S ribosomal protein S6 kinase (p70S6 kinase) and 4E-binding protein 1 (4E-BP1) [41,46]. While it is well established that mTORC1 directly suppresses autophagy, mTORC2 may also regulate autophagy via Akt or PKC.

mTORC1 activity depends on the nutrient status of the cells. A direct role for mTORC1 in autophagy came from the studies that showed that mTORC1 phosphorylates and regulates proteins involved in autophagosome formation. In yeasts, TOR inhibits the association between ATG1 (ULK1 in humans) and ATG13 by hyperphosphorylating ATG13, thereby decreasing its affinity for ATG1 [47,48]. Recent studies suggest that when nutrient is abundant, active mTORC1 inhibits autophagosome formation by associating with the ULK1-ATG13-FIP200 complex and phosphorylating ULK1 and Atg13 [12,49-52]. Inhibition of mTORC1 by rapamycin or starvation results in dephosphorylation of ULK1 and initiation of autophagy. mTORC1 has also been shown to play a role in the termination of autophagy and lysosomal homeostasis [53]. Degradation of autolysosomal products during prolonged starvation results in reactivation of mTOR [53]. Active mTOR inhibits autophagy and allows formation of mature functional lysosomes through an unknown mechanism [53].Since p70S6 kinase or S6K is a downstream target of mTORC1, it is considered a negative regulator of autophagy [54]. Consistent with this hypothesis, S6K activity correlates with autophagy suppression [54]. Several studies, however, suggest that S6K promotes rather than inhibits autophagy. Scott *et al* demonstrated that in the *Drosophila* fat body, starvation induces autophagy that peaks within hours following nutrient withdrawal and then reaches low levels over extended periods of starvation so as to promote only survival and avoid autophagic cell death [55]. The study also revealed that TOR inhibited autophagy independent of dS6K and that dS6K was in fact required for starvation-induced autophagy. The inhibition of S6K by resveratrol was also shown to impair the autophagic response [56] and S6K positively regulated 6-thioguanine-induced autophagy in colorectal cancer cells [57].

The mechanism by which S6K promotes autophagy may involve synthesis of proteins that participate in autophagosome formation and maturation [58,59] or modulation of pathways that directly regulate autophagy. For example, activation of S6K inhibits insulin/IGF signaling via a negative feedback loop that involves phosphorylation of insulin receptor substrate [60-62]. While autophagy is a beneficial process that recycles cellular contents for survival, excessive self-eating can cause cell death. Thus, placing a positive regulator of autophagy under the control of mTOR ensures that extended starvation leading to mTOR inhibition prevents unrestrained autophagy, which causes cell death [13]. The negative role of mTOR and the positive role of S6K in autophagy may provide a balance in the levels of autophagy over extended periods of nutrient deprivation.

Another downstream target of mTOR involved in regulating excessive autophagy is death-associated protein 1 (DAP1) [63]. DAP1 is a cytosolic protein implicated in cell death in response to interferon-gamma [64]. It is functionally silenced in growing cells by mTOR-dependent phosphorylation [63]. Under amino acid deprivation, decrease in mTOR-mediated DAP1 phosphorylation restores the anti-autophagic function of DAP1. Thus, DAP1 reactivation in the absence of mTOR function prevents excessive autophagy during nutrient-deprived condition. mTORC1 can also regulate autophagy by unknown mechanisms that are rapamycin-insensitive [65]. Finally, mTORC2 can negatively regulate autophagy indirectly through Akt. Activation of Akt results in phosphorylation and inactivation of FoxO3 [66,67], which regulates the expression of autophagy-related genes, such as LC3 and Bnip3 [66,67]. Phosphorylation of FoxO3 by Akt results in its nuclear export and cytoplasmic sequestration by 14-3-3 proteins, thereby preventing the expression of its target genes [67-69].

## 3. AMPK

As part of the energy-sensing cascade, mTORC1 is also regulated by the AMP-dependent protein kinase (AMPK). AMPK is activated in response to stress and low cellular ATP levels and phosphorylates TSC2 (tuberous sclerosis complex-2) and raptor, resulting in inhibition of mTORC1 activity [70,71]. Thus, AMPK functions to shut down mTORC1-mediated anabolic processes under low energy conditions and induces autophagy to generate products for ATP synthesis. Until recently, the sole mechanism by which AMPK activates autophagy was thought to be through the inhibition of mTORC1 and hence indirectly promoting autophagosome formation. Recent reports, however, suggest a more direct role for AMPK in autophagy regulation. Similar to mTORC1, AMPK was shown to interact with ULK1 [72]. Two independent groups demonstrated that AMPK directly phosphorylates ULK1 although there are controversies regarding the sites of phosphorylation [49,73]. Also, the interaction between ULK1 and AMPK was not affected by glucose starvation. However, mTORC1-mediated phosphorylation of ULK1 at Ser757 disrupted the ULK1-AMPK interaction [49]. Thus, AMPK ensures the induction of autophagy under low energy conditions by dual regulation of mTORC1 and ULK1. Activation of AMPK also leads to the nuclear accumulation of FoxO3a and upregulation of autophagy genes [74].

## 4. PI3K and Akt

The phosphoinositide 3-kinases are enzymes that catalyze the phosphorylation of the 3′ position hydroxyl group of the inositol ring in phosphatidylinositol (PtdIns). Based on structure, function and substrate specificity, the PI3Ks have been classified into three classes [75]. Class I PI3Ks are often activated in response to growth factors and catalyze the production of phosphatidylinositol-3-phosphate PtdIns(3)P, PtdIns(3,4)P2 and PtdIns(3,4,5)P3. These second messengers enhance membrane recruitment of pleckstrin-homology (PH) domain-containing proteins such as phosphoinositide-dependent kinase-1 (PDK1) as well as its substrate Akt/protein kinase B, facilitating Akt activation [76]. Little is known about the function of class II PI3K. It is believed to play a role in processes such as cell migration and vascular smooth muscle contraction [77,78]. The sole member of class III PI3K is Vps34 (vacuolar protein sorting 34). It was originally identified as a mediator of vesicular trafficking of proteins that have PtdIns(3)P binding domain to recruit them to intracellular membranes such as endosomal and lysosomal membranes [79].

The class III PI3K, Vps34 plays an important role in mediating autophagosome formation. It directly interacts with beclin 1 [80] and promotes the formation of the autophagosome vesicle [20]. It facilitates the recruitment of proteins with phospholipid-binding domain such as the WD40-repeat proteins hWIPI-1alpha [81] and hWIPI2 [82], the human orthologs of the yeast autophagy gene Atg18, to the early autophagosome structure. Following amino acid deprivation, hWIPI-1alpha was shown to colocalize with the autophagosome marker MAP1LC3 (LC3) [81]. Studies in yeast showed that the phospholipid-binding domain in Atg18 binds PtdIns(3)P and is involved in the generation of the pre-autophagosome structures [83]. The presence of WD40-repeats in hWIPI may facilitate the formation of multi-protein structures and play a scaffolding role in building the autophagosome [81,84]. Recently, hWIPI2 was reported to play a role in LC3 lipidation [82].

While class III PI3K directly participates in autophagy, class I PI3K can regulate autophagy indirectly via Akt and mTORC1. It mediates growth factor and insulin signaling and leads to the activation of Akt via PDK1. Once activated, Akt phosphorylates and inhibits TSC2, which acts as a GTPase-activating protein (GAP) for ras homolog enriched in brain (Rheb), a GTP binding protein. GTP-bound Rheb leads to the activation of and signaling through the mTORC1 complex resulting in protein synthesis, cell growth and inhibition of autophagy [85]. Thus, the growth signals from PI3K/Akt and autophagy regulation are integrated by mTOR.

hVps34 has also been shown to positively regulate the activity of mTORC1 in response to amino acids [86]. Amino acid stimulation leads to the increased activity of hVps34. Although mTORC1 was insensitive to growth factors in the absence of TSC1/TSC2, it remained responsive to the levels of amino acids [87,88]. This observation suggests that amino acids activate mTOR through a pathway distinct from the PI3K/Akt/TSC pathway. However, wortmannin, a class I PI3K inhibitor prevented the amino acid-mediated S6K1 activation [87]. This led to the identification of a novel target of wortmannin, hVps34, which caused amino acid-dependent activation of S6K1 [87,89]. While the role of hVps34 in mTOR activation in response to amino acids is being addressed, the precise mechanism by which this occurs is poorly understood.

The observation that hVps34 positively regulates autophagosome formation as well as mTORC1, a negative regulator of autophagy, is counterintuitive. An explanation for this paradoxical role is that its binding partners such as beclin 1 and its localization determine specific functions in response to starvation or amino acids. For example, Kihara *et al.* [20] demonstrated that two distinct Vps34 containing complexes participate in autophagy versus carboxypeptidase Y sorting.

## 5. Mitogen-Activated Protein Kinase (MAPK)

Mitogen activated protein kinases (MAPKs) are serine/threonine kinases that mediate responses to various extracellular stimuli. Extracellular signals such as growth factors lead to a sequential phosphorylation cascade that ultimately results in MAPK activation. Once activated, MAPKs lead to phosphorylation-dependent activation of other kinases and transcription factors. The four categories of MAPKs are extracellular signal-regulated kinase (ERK)/mitogen-activated protein kinase (MAPK), p38, c-Jun N-terminal kinase (JNK)/stress-activated protein kinase (SAPK) and big MAP kinase (BMK) [90]. The best-studied MAP kinases are ERK, p38 and JNK. While ERK is activated in response to proliferative signals, p38 and JNK are activated in response to various stresses.

### 5.1. ERK

ERK1 (p44) and ERK2 (p42) are two isoforms of ERK that are activated downstream of Ras in response to extracellular cues. Ras binds to and activates Raf and initiates activation of MAPK signaling cascade. Raf activates MEK which in turn phosphorylates and activates ERK1/2 [91]. ERK controls cell proliferation, migration, differentiation and cell death [92]. ERK not only plays an important role in regulating cell death by apoptosis but has also been implicated in autophagy [93].

One of the earliest studies used pharmacological inhibitors of ERK to demonstrate that it mediates starvation-induced autophagy by phosphorylation and consequent activation of GAIP (Gα interacting protein) in human colon cancer cells [94]. ERK has been shown to induce autophagy in response to a number of anti-tumor/cytotoxic agents, such as soyasaponins in colon cancer cells [95], capsaicin in breast cancer cells [96] and cadmium in mesangial cells [97,98]. Inhibition of ERK was associated with a decrease in autophagy and increased cellular sensitivity to tumor necrosis factor-α (TNF) in breast cancer MCF-7 cells [99]. ERK was shown to promote TNF-induced autophagy in mouse fibroblast L929 cells by activating p53 [100]. Activation of ERK by the transformation of human mesenchymal stem cells by H-ras [101], overexpression of TrkA (tropomyosin-related kinase A) in glioblastoma cells [102] and overexpression of mutant LRRK2 (leucine rich repeat kinase 2) in neuronal cells [103] was associated with increase in autophagy.

Recent studies suggest that ERK regulates the maturation of autophagic vacuoles. Activation of ERK has been associated with the formation of large cytoplasmic vacuoles [104-106]. Lindane, a carcinogen, caused formation of large vacuoles that displayed arrested autolysosomes, incapable of degrading the autophagic contents in Sertoli cells [107]. This vacuolation was independent of mTORC1 activity but was dependent on sustained activation of ERK [107]. The authors speculated that ERK facilitates malignant growth by inhibiting tumor suppressive function of autophagy [107]. In contrast, capsaicin-induced autophagy in breast cancer cells required ERK activity at the maturation step since inhibition of ERK resulted in increased LC3-II indicative of degradation blockade [96]. Non-canonical activation of MEK/ERK can also promote autophagy by modulating beclin 1 expression. It has been reported that activation of MEK/ERK downstream of AMPK leads to disassembly of mTORC1 and mTORC2, and an increase in beclin 1 expression [108]. While acute activation of MEK/ERK leads to inhibition of either mTORC1 or mTORC2 and moderate increase in beclin 1 expression resulting in cytoprotective autophagy, sustained activation of MEK/ERK causes inhibition of both mTORC1 and mTORC2, marked increase in beclin 1 and cytodestructive autophagy [108].

Important insights into the role of ERK in autophagy/mitophagy came from studies in neurodegenerative disorders [109-112]. ERK appears to play an important role in bulk as well as mitochondrial autophagy. Overexpression of ERK2 was sufficient to induce autophagy as well as mitophagy in glioblastoma cells although mitophagy correlated more closely with ERK2 activity as compared to general autophagy [109]. Interestingly, mitochondrial ERK is also known to protect cancer cells from apoptosis [113]. It is conceivable that mitochondrial ERK causes chemoresistance in cancer cells via induction of mitophagy.

### 5.2. p38

p38 MAPK family comprises of four isoforms p38α, -β, -γ and -δ [114]. p38α is the best studied isoform in the family. Most studies suggest a tumor suppressive role for p38, such as inhibition of cell cycle progression, induction of apoptosis and terminal differentiation [115]. However, p38 has also been implicated in tumor progression by promoting invasion, angiogenesis and inflammation [115]. Recent studies suggest that p38 also regulates autophagy although there are controversies if it promotes or inhibits autophagy [116].

In yeast, the p38 homolog Hog1 was shown to positively regulate autophagy in response to osmotic stress [117,118] and ER stress [119]. Chronic ER stress caused phosphorylation of Hog1, which enhanced the stability of Atg8, thereby promoting autophagy [119]. Activation of p38 was associated with induction of autophagy by polygonatum cyrtonema lectin (PCL) in human melanoma cells [120], MCP-1 (monocyte chemotactic protein-1) in cardiomyoblast [121], silibinin in fibrosarcoma cells [122], bromelain in breast cancer cells [123], oridonin in HeLa cells [124] and resveratrol in hepatocellular carcinoma cells [125]. The induction of autophagy by p38 was often accompanied by an increase in Atg proteins, such as beclin 1 and Atg5 [124-127]. In several studies, the tumor suppressor protein p53 was shown to be involved in the autophagy induction by p38 [120-122]. Phosphorylation of p53 at Ser392 by p38 enhanced its transcriptional activity causing increased expression of beclin 1 [120]. ER stress-induced beclin 1 expression and induction of autophagy also correlated with increased p38 phosphorylation/activation [126].

p38 was also shown to negatively regulate autophagy. Inhibition of p38 in colon cancer cells [128] and myelogenous leukemic K562 cells [129] was associated with increase in beclin 1 and induction of autophagy. Many of the inhibitors used in these studies have multiple targets and therefore it is difficult to discern the contribution of p38 in autophagy in these studies. A recent report, however, demonstrated that p38 inhibits autophagy by competing with transmembrane protein mAtg9 for binding to p38 interacting protein (p38IP) [130]. The cycling of mAtg9 between trans-Golgi network (TGN), endosomes and growing autophagosome is required for autophagy and is achieved by its binding with p38IP [116].

### 5.3. JNK

JNK family is comprised of three members JNK1, -2 and -3 [90,115]. While JNK1 and -2 are expressed ubiquitously, JNK3 is primarily expressed in the brain [90,115]. JNK is activated downstream of various stress stimuli, such as heat shock, osmotic shock, ultraviolet irradiation and cytokines [131-134] and mediates its diverse function by acting on downstream targets that include transcription factors, such as AP1, Elk1, ATF2 and p53 as well as antiapoptotic protein Bcl-2 [115].

JNK has been implicated in the induction of autophagy by various stimuli, including starvation [135], cytokine stimulation [136], T-cell receptor activation [135], neuronal excitotoxic stimuli [137] and ER stress [138]. One mechanism by which JNK contributes to autophagy involves phosphorylation of the antiapoptotic protein Bcl-2 [139-142]. In nutrient-rich conditions, binding of Bcl-2 to beclin 1 inhibits autophagy [143]. Phosphorylation of Bcl-2 by JNK causes its dissociation from beclin 1, resulting in induction of autophagy [144]. Phosphorylation of Bcl-2 by JNK has also been implicated in the induction of apoptosis [145]. Bcl-2 not only inhibits autophagy by interacting with beclin 1 but it can also inhibit apoptosis by sequestering the proapoptotic protein Bax [146,147]. It has been reported that acute nutrient starvation although sufficient to dissociate beclin 1 from Bcl-2-beclin 1 complex, cannot disrupt Bcl-2-Bax complex [145]. Sustained nutrient starvation that results in high levels of Bcl-2 phosphorylation is required to dissociate Bax from Bcl-2. It was speculated that rapid Bcl-2 phosphorylation initially promotes cell survival by inducing autophagy by releasing beclin 1 which has low affinity for Bcl-2 but when autophagy fails to keep cells alive, Bcl-2 phosphorylation triggers apoptosis by dissociating Bax from the complex [145]. Thus, the levels of JNK1-mediated Bcl-2 multisite phosphorylation would determine cell fate by differential regulation of autophagy *vs.* apoptosis [144].

Besides its role in posttranslational modification of proteins that regulate autophagy, JNK can also regulate the expression of beclin 1 transcript via phosphorylation of c-Jun [123,148,149]. However, increased intracellular levels of hydrogen peroxide induced non-canonical autophagy which was beclin 1 independent but required JNK-mediated activation of Atg7 [150]. JNK has been shown to induce upregulation of p62, Atg5 and Atg7 in response to resveratrol, oxidative stress and oncogenic transformations [150-152].

JNK can also promote autophagy via p53 in several cell types, including human colon carcinoma and fibrosarcoma [100,122,153,154]. p53 is phosphorylated by various kinases including p38 and JNK [155] and phosphorylation of p53 results in its stabilization and activation. p53 can promote autophagy by upregulation of several pro-autophagy genes, such as AMPK, Bnip3 (Bcl-2 interacting protein 3) and DAPK-1 (death associated protein kinase-1) [156].

In contrast to the above mentioned pro-autophagic role of JNK, a recent study demonstrated that targeted deletion of *JNK 1*, *JNK2* and *JNK3* in neurons increased autophagy [157]. The increase in autophagy in neurons from triple knockout mice was independent of mTORC1 but was associated with an increase in FoxO1 transcription factor and its transcriptional target Bnip3, a BH3-only member of the Bcl-2 family. In neurons, beclin 1 complexed with Bcl-xl and not Bcl-2. Bnip3 competes with beclin 1 for binding to Bcl-xL via BH3 domain, thereby causing release of beclin 1 and induction of autophagy. Since JNK3 is predominantly expressed in brain, it remains to be seen if ablation of *JNK3* alone is sufficient to increase autophagy to provide neuroprotection. Moreover, future studies should determine if different isoforms of JNK have distinct role in cancer *versus* neurodegenerative disorders.

## 6. Protein Kinase C

Protein kinase C (PKC) is a family of phospholipid-dependent serine/threonine kinases that regulate diverse cellular functions. Based on the structural features and cofactor requirements, PKC members are classified as conventional (PKCα, -βI, -βII and -γ), novel (PKCδ, -ε, -η and -θ) and atypical (PKCζ and -ι) PKCs [158]. While conventional PKCs require both Ca^2+^ and diacylglycerol (DAG) for their activity, novel PKCs are Ca^2+^-independent but DAG-dependent whereas atypical PKCs are independent of both Ca^2+^and DAG. Conventional and novel PKCs are the receptors for tumor-promoting phorbol esters, which are potent activators of PKCs and can substitute for the physiological activator DAG [159]. PKCs have been linked with both tumor promotion and tumor suppression [160,161]. The involvement of PKCs in apoptosis is also well established. PKCδ was first identified as a substrate for caspase-3 and proteolytic activation of PKCδ was directly linked with apoptosis [162,163]. Subsequently, several members of the PKC family, including PKC-ε, -θ and -ζ were identified as substrates for caspases [164-166]. In contrast to PKCδ and -θ, PKCε and -ζ are believed to function as antiapoptotic proteins [162,164-169]. The involvement of PKCs in autophagy has just begun to emerge.

As with apoptosis, PKCδ is the first PKC isozyme shown to play an important role in autophagy. PKCδ suppressed autophagy in pancreatic ductal carcinoma cells by increasing the expression of tissue transglutaminase 2 (TG2) [170] which negatively regulates autophagy [171]. In contrast, PKCδ promoted autophagy in rat parotid epithelial cells during acute hypoxic stress by activating JNK [139], which phosphorylates Bcl-2 and dissociates it from beclin 1 [172,173]. Chronic hypoxic stress, however, attenuated beclin 1-dependent autophagy [174]. The authors speculated that PKCδ protects cells from acute stress by inducing autophagy but cleavage of PKCδ by caspase-3 during chronic stress relieves the protection against cell death by autophagy and irreversibly commits cells to apoptosis [139]. The involvement of PKCδ/JNK pathway in autophagy was recently corroborated by Shahnazari *et al.* [175] who reported that PKCδ as well as its downstream targets JNK and NADPH oxidase are indispensible for antibacterial autophagy [175]. Safingol-induced autophagic cell death was associated with downregulation of PKCδ and -ε [176]. Thus, whether or not PKCδ will promote or protect against autophagy depends on the duration of the stress as well as cellular context.

PKCθ, another member of the novel PKC family, was shown to influence endoplasmic reticulum (ER) stress-induced autophagy but had no effect on amino acid starvation-induced autophagy [177]. ER stress inducers caused calcium-dependent phosphorylation of PKCθ and translocation of PKCθ to LC3-II containing vesicles in the cytoplasm. siRNA mediated knockdown of PKCθ or phosphorylation-defective mutant of PKCθ inhibited ER stress-induced autophagy [177]. It is not clear how PKCθ regulates autophagy. Since activation of novel PKCs is independent of Ca^2+^ and phosphorylation of PKCs primes them for activation, it is conceivable that an alteration in PKCθ localization is responsible for ER stress-induced autophagy. Recently, it has been reported that PKC can inhibit autophagy induced by starvation or rapamycin in a PI3K-independent manner [178]. Several recombinant PKCs, including PKCθ, were shown to directly phosphorylate LC3 at Thr6 and Thr29 sites although conventional PKCs (with the exception of PKCβI) were most effective. However, mutation of these Thr residues to non-phosphorylatable Ala had little effect on starvation-induced autophagy. It remains to be seen if phosphorylation of LC3 or some other proteins in the autophagosome by PKCθ influences ER stress-induced autophagy.

Conventional PKCα has also been implicated in autophagy. Dephosphorylation of PtdIns(4,5)P_2_ (phosphatidyl inositol-4,5-bis phosphate) by phosphatases or masking the lipid with PH domain led to mitochondrial fission and mitophagy in different cell lines [179]. Additionally, loss of mitochondrial PtdIns(4,5)P_2_ was associated with an increase in starvation-induced autophagy. However, co-expression of mitochondrially targeted PKCα could rescue cells from mitophagy and cell death caused by PtdIns(4,5)P_2_ unavailability [179]. Previously it has been shown that mitochondrial PKCα inhibits apoptosis by phosphorylating Bcl-2 [180]. Thus, PKCα appears to play a major role in maintaining the mitochondrial integrity. A recent study, however, demonstrated that mammalian PKCα facilitates the insertion of active form of Bax into the outer mitochondrial membrane of yeast and this was accompanied by an increase in apoptosis as well as autophagy [181]. PKCα kinase activity was not required for the translocation and insertion of active Bax into the mitochondria. PKCα has been shown to function both as anti- and pro-apoptotic protein depending on the cell type. It remains to be seen if kinase-independent function of PKCα is necessary for its proautophagic function in higher organisms.

In addition to the involvement of specific PKCs in autophagy, several studies have employed pharmacological activators or inhibitors of PKCs to establish their role in autophagy. PKC was shown to positively regulate oridonin-induced autophagy in HeLa cells via Raf1/JNKpathway [182] and acadesine (AICAR, 5-Aminoimidazole-4-carboxamide-1-beta-D-ribofuranoside)-induced autophagic cell death in chronic myelogenic leukemia cells [183]. Caution should be exercised when pharmacological PKC modulators are used unless substantiated by molecular approaches. For example, the PKCδ inhibitor rottlerin was shown to induce autophagy in colon cancer cells via PKCδ-independent mechanisms [184].

## 7. Kinases Involved in Autophagy Regulation During ER Stress

Autophagy is activated in response to multiple stresses such as pathogen infection, nutrient deprivation, hypoxia and endoplasmic reticulum (ER) stress [4]. Several kinases have been reported to mediate autophagy in response to these stress signals, especially ER stress. For example, the accumulation of unfolded and misfolded proteins in the endoplasmic reticulum (ER) lumen leads to a cellular stress response called the unfolded protein response (UPR). ER stress is a potent inducer of macroautophagy, possibly to facilitate the removal of unfolded or misfolded proteins [185]. Several ER stress-activated kinases such as IRE1, Ca^2+^/calmodulin-dependent kinase kinase- beta (CaMKKβ), death associated protein kinase (DAPK) and PERK have been associated with ER stress-induced autophagy. Once activated, these kinases can ultimately lead to the upregulation of autophagy-related genes and inhibition of autophagy suppressors.

One of the first proteins activated in response to UPR is the inositol-requiring enzyme 1 (IRE1) [186]. In yeast, activated IRE1 leads to the maturation of the Hac1 (homologous to activating transcription factor (ATF)/CREB1) mRNA [187] and subsequent transcriptional upregulation of genes involved in protein folding and autophagy genes such as ATG5, 7, 8 and 19 [188]. In mammalian cells, IRE1 was required for the accumulation of LC3-II positive vesicles in response to ER stress inducers such as tunicamycin and thapsigargin [138].

As a part of UPR, PERK directly phosphorylates eukaryotic initiation factor 2α (eIF2α) to block translation initiation. Recently, it has been shown that PERK-mediated phosphorylation of eIF2α is also required for LC3 conversion during ER stress-induced autophagy [189,190]. It can also increase the expression of Atg5 to promote autophagy [191]. ER stress also leads to the release of Ca^2+^ stored in the ER to the cytoplasm, causing activation of various Ca^2+^-dependent kinases. Studies that employed RNA interference and pharmacological inhibitors showed that Ca^2+^-induced autophagy was dependent on the Ca^2+^/calmodulin-dependent kinase kinase-beta (CaMKKβ), which is an upstream activator of AMPK resulting in mTORC1 inhibition and autophagy induction [192,193].

Death-associated protein kinase 1 (DAPK1) is another Ca^2+^/calmodulin-regulated kinase that plays an important role in ER stress-induced autophagy. Recently, Zalckvar *et al.* showed that DAPK1 mediates induction of autophagy by phosphorylating beclin 1 [194]. Phosphorylated beclin 1 dissociates from Bcl-X_L_ and mediates autophagosome formation [194]. DAPK1 also associates with microtubule associated protein 1B (MAP1B) and this complex is supposed to be required for autophagosome formation [195,196].

## 8. Conclusions

The phenomenon of autophagy was described nearly half a century ago. However, research into the molecular mechanisms and regulation of autophagy has taken serious form only during the past decade. There is a growing interest in modulating autophagy for cancer therapy. While excessive autophagy is recognized as a type of cell death, its role in protecting cancer cells from stress leads to the debate as to whether autophagy should be inhibited or activated for cancer therapy. Addressing this question would require a thorough understanding of the regulation of autophagy so that appropriate proteins are chosen as targets.

**Figure 1.**
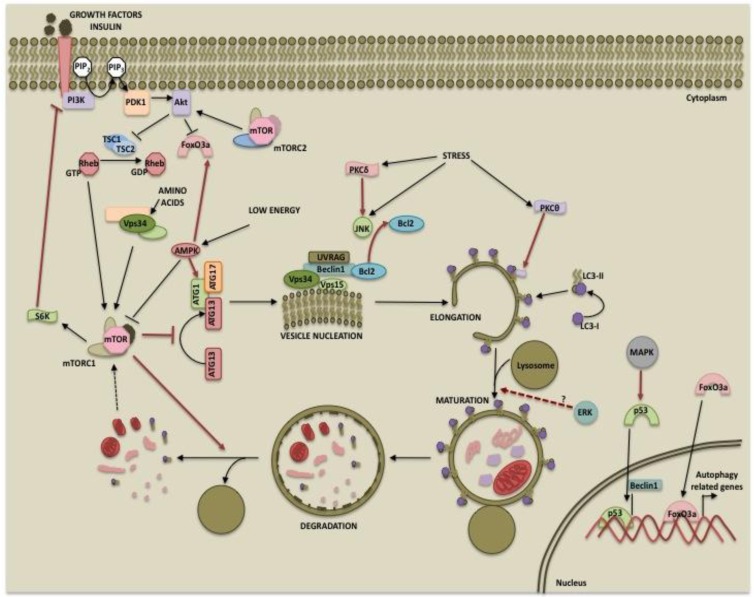
The regulation of autophagy by several cellular kinases. PI3K: phosphoinositide 3-kinases; PDK1: phosphoinositide-dependent kinase-1; mTOR: mammalian target of rapamycin; TSC: tuberous sclerosis complex; AMPK: AMP-dependent protein kinase; ERK: extracellular signal-regulated kinase; PKC: protein kinase C.

Posttranslational modifications of a protein often determine its function. It is now evident that autophagy can be regulated by kinases at multiple steps such as autophagosome inception, vesicle maturation, termination and autophagy-related gene expression as depicted in Figure 1. This suggests that the process can be modulated at multiple steps for therapy. While the PI3K/mTOR pathway primarily inhibits autophagy, the role of the MAPK pathway and PKCs in autophagy may depend on the cellular context and inducers used. Also, there is extensive cross-talk among these pathways. For example, while both the PI3K and MAPK pathways are activated by receptor tyrosine kinases, the two pathways may either be antagonistic to each other or converge at the level of mTOR signaling. These pathways are activated in several cancers and play important roles in tumorigenesis and cancer progression. Differential regulation of autophagy by these two pathways could provide a tighter regulation to maintain it at levels sufficient to promote cell survival and prevent excessive self-eating and cell death. While one pathway initiates autophagy, the other pathway might be instrumental in its completion or can provide inhibitory signals at a later time to prevent excessive catabolism. Hence, the ultimate outcome will depend upon factors such as which autophagy regulatory pathways prevail, the extent of autophagy occurring and the presence of feedback control mechanisms. Our current knowledge and future work in the area of autophagy regulation should aid in designing better therapeutic approaches for cancer.
